# Biomaterial-Mediated Modification of the Local Inflammatory Environment

**DOI:** 10.3389/fbioe.2015.00067

**Published:** 2015-05-15

**Authors:** Shane Browne, Abhay Pandit

**Affiliations:** ^1^Network of Excellence for Functional Biomaterials (NFB), National University of Ireland, Galway, Ireland

**Keywords:** biomaterials, foreign body response, inflammation, drug delivery, controlled release

## Abstract

Inflammation plays a major role in the rejection of biomaterial implants. In addition, despite playing an important role in the early stages of wound healing, dysregulated inflammation has a negative impact on the wound healing processes. Thus, strategies to modulate excessive inflammation are needed. Through the use of biomaterials to control the release of anti-inflammatory therapeutics, increased control over inflammation is possible in a range of pathological conditions. However, the choice of biomaterial (natural or synthetic), and the form it takes (solid, hydrogel, or micro/nanoparticle) is dependent on both the cause and tissue location of inflammation. These considerations also influence the nature of the anti-inflammatory therapeutic that is incorporated into the biomaterial to be delivered. In this report, the range of biomaterials and anti-inflammatory therapeutics that have been combined will be discussed, as well as the functional benefit observed. Furthermore, we point toward future strategies in the field that will bring more efficacious anti-inflammatory therapeutics closer to realization.

## Introduction

The inflammatory response is an essential part of the healing process (Martin and Leibovich, 2005; Li et al., 2007). Inflammation is initiated by necrosis and tissue injury, through the recognition of damage associated molecular patterns (DAMPs). DAMPs that trigger inflammation include intracellular proteins and nucleic acids released by dying cells, and extracellular matrix (ECM) fragments such as low molecular weight hyaluronic acid (HA) (Kataoka et al., 2014). In addition, stores of inflammatory cytokines including IL-1α and IL-33 present in cells are released following necrotic cell death (Chen and Nunez, 2010). Inflammation is required to remove necrotic and apoptotic cells, cleaved ECM molecules, and to initiate subsequent angiogenesis and tissue repair (Jiang and Liao, 2010). In fact, inflammatory cells have been shown to play a role in regeneration (Kim et al., 2013). However, excessive and chronic inflammation leads to the formation of a hostile environment for regeneration and repair, resulting in further cell death. Excessive inflammation and ECM remodeling lead to the formation of a fibrotic scar through the upregulation of matrix metalloproteinases (MMPs) and increased deposition of collagen type I and III (Dobaczewski and Frangogiannis, 2008). This is typically characterized by increased neutrophils infiltration and pro-inflammatory macrophage retention. This amplifies the pro-inflammatory cytokine response, along with MMP activity and the presence of radical oxygen species (ROS). It has been shown that improved wound healing occurs following a lesser inflammatory response in fetal wound healing (Redd et al., 2004), with a reduced expression of pro-inflammatory cytokines, transforming growth factor-beta (TGF-β), and overexpression of interleukin-10 (IL-10) (Lo et al., 2012). A similar effect has been observed in fetal myocardium, with reduced inflammation allowing for complete functional restoration (Herdrich et al., 2010). Furthermore, reduction in inflammation has been shown to promote mesenchymal stem cell (MSC)-mediated bone tissue regeneration (Liu et al., 2011; Chang et al., 2013). While scarless healing cannot be completely attributed to the absence of inflammation, it does present evidence that a reduced inflammatory response can result in a more favorable outcome.

Thus, strategies to reduce inflammation can prove to be of benefit to treat conditions in which excessive inflammation causes damage to the tissue, or when inflammation becomes chronic.

## Biomaterials to Modulate Inflammation

Implanted biomaterials can cause an inflammatory response, with the level of this response dependent on the material of choice along with the site in the body into which it is implanted (Luttikhuizen et al., 2006a,b; Anderson et al., 2008). This is known as the foreign body response (FBR) and is the response of the host to the implant. Following implantation, a biomaterial acquires a layer of host proteins that is associated with the surface chemistry of the material. This occurs before any interaction with host cells, and governs the type of cells that interact and their phenotypes. Material properties and, particularly, surface chemistry have an effect on protein deposition on the surface. The proteins that typically absorb on the surface include fibrinogen, albumin, and fibronectin. These proteins form a provisional matrix composed mostly of fibrin around the implant. This matrix acts like a thrombus and initiates a wound healing-like inflammatory response. Inflammatory cells such as neutrophils and macrophages are attracted by the build-up of chemokines and other chemoattractants in the provisional matrix. Therefore, the choice of biomaterial is of the utmost importance, with an obvious preference for materials that cause a minimal acute response. Typically, anti-inflammatory strategies using biomaterials have involved loading of therapeutics into biomaterial systems, with therapeutic release *in vivo* aiding to alleviate inflammation. These anti-inflammatory signals are composed of anti-inflammatory drugs, proteins, or nucleic acids, while the delivery of stem cells has also been shown to result in a reduced inflammatory response. In addition, a number of naturally occurring biomaterials have intrinsic anti-inflammatory signals. These include high molecular weight HA (Nakamura et al., 2004; Hirabara et al., 2013) and chitosan, which have ROS-scavenging properties (Je and Kim, 2006). However, in the case of most materials, loading of anti-inflammatory therapeutics is necessary to modulate the inflammatory microenvironment.

**Figure 1:**
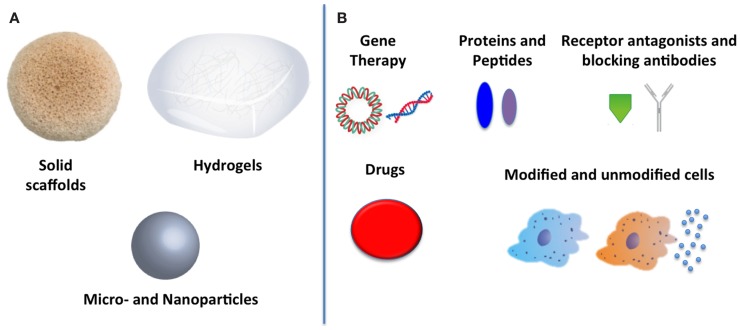
**Biomaterials to reduce inflammation: (A) biomaterial forms used to deliver therapeutics and (B) anti-inflammatory therapeutics that have been delivered**.

A wide variety of therapies to reduce inflammation exist, from gene therapy to receptor blocking antibodies, protein delivery, and cell therapy (see Figure 1B). However, as yet, no outstanding candidate has emerged that can convincingly reduce inflammation in all situations. Each therapy has associated advantages and disadvantages. However, the main drawback associated with most therapies is inadequate efficacy as a result of an insufficient local concentration. This may be due to minimal localization at the appropriate site of action when administered systemically, or as a result of clearance and destruction by inflammatory cells when administered locally. One route to address this concern is by the use of biomaterial systems as reservoirs of therapeutics to locally deliver and sustain effective concentrations for a prolonged period of time. Protein and gene delivery through scaffolds holds much promise to produce efficacious therapies (O’Rorke et al., 2010; Monaghan and Pandit, 2011; Censi et al., 2012). Natural biomaterials are capable of loading and releasing therapeutics through MMP-mediated biodegradation, with collagen in particular standing out for its usefulness (Browne et al., 2013). However, synthetic materials may allow for increased control over degradation and release kinetics of therapeutics, with the caveat that the material itself and its degradation products must cause a minimal response when implanted *in vivo*.

A further consideration that must be taken into account is the form the biomaterial system takes. Three typical structures that biomaterials can take are solid scaffolds, hydrogels, and particles, shown in Figure 1A. The choice of biomaterial structure is dependent on a number of factors, primarily the tissue being targeted and also the nature of the anti-inflammatory therapeutic incorporated. For example, solid scaffolds require surgery to implant, and thus are more suitable for wound healing applications in which they may be applied to an open wound. In contrast, *in situ* gelling hydrogels and particles can typically be delivered in a minimally invasive manner using a syringe without the need for open surgery. Thus, hydrogels and particles are suitable for delivery directly into tissues such as muscle and the myocardium. However, in terms of loading, they differ. Particles are most suited for protein, gene, and drug delivery (Sehgal and Srinivasan, 2009; Browne et al., 2012; Kraskiewicz et al., 2013). Hydrogels may not only be used for protein, gene, and drug delivery but also for cells. Hydrogels can be designed to provide a microenvironment that can be tuned to protect implanted cells, promoting cell survival, and improving function (Seliktar, 2012). Meanwhile, solid scaffolds have also been loaded with proteins, nucleic acids, and drugs, in addition to acting as matrices to enhance cell transplantation (Thevenot et al., 2010; Holladay et al., 2011; Hortensius et al., 2015). Thus, the choice of biomaterial form is dependent on the tissue into which it will be implanted, and the anti-inflammatory agent being delivered. The main biomaterial approaches to reducing inflammation will be discussed in terms of the type of biomaterial structure used.

### Solid scaffolds

The incorporation of stromal cell-derived factor-1 alpha (SDF-1α) into a poly (lactic-co-glycolic acid) PLGA scaffold reduced the inflammatory response when implanted into the subcutaneous space in mice (Thevenot et al., 2010). While SDF-1α is typically associated with increased angiogenesis, it can also exert anti-inflammatory effects through its mobilization and homing effect on stem cells (Ceradini et al., 2004; Ceradini and Gurtner, 2005). Thus, it was shown that incorporation of SDF-1α in PLGA scaffolds reduced the inflammatory tissue response through an increase in autologous stem recruitment to the implant site. Furthermore, a reduction in pro-inflammatory cytokines was detected, with reduced expression of a number of key mediators including interleukin-1 alpha (IL-1α), interleukin-6 (IL-6), and tumor necrosis factor alpha (TNF-α), while there was an increase in vascular endothelial growth factor (VEGF) expression.

Modification of the inflammatory response to a carbodiimide crosslinked collagen scaffold was achieved by codelivery of a plasmid encoding IL-10 (*p*IL-10). Treatment with the *p*IL-10 reduced the inflammatory response to the implanted collagen scaffolds in a subcutaneous model, with a reduction seen in infiltrating macrophages (ED1 positive cells). However, a subsequent reduction in vascularization was also observed (van Putten et al., 2009). A collagen scaffold with MSCs incorporated was further functionalized with *p*IL-10 polyplexes in an effort to reduce inflammation in an intramuscular model, and to promote the survival of the MSCs. It was observed that *p*IL-10 polyplex treatment reduced inflammation and increased MSC survival (Holladay et al., 2011). When this system was implanted in a rodent model of myocardial infarction (MI), reduced inflammation was detected along with functional recovery of the heart, in terms of improved ejection fraction (Holladay et al., 2012). Furthermore, a change in macrophage phenotype was detected. Macrophages were seen to change from a classically activated, pro-inflammatory phenotype in control groups to an alternatively activated, anti-inflammatory phenotype following treatment with *p*IL-10 polyplexes and MSCs. Macrophage phenotype has been shown to be a key component of the inflammatory response to implanted biomaterials (van Putten et al., 2013; Spiller et al., 2014), and thus strategies to control the phenotype of macrophages may prove vital to modify inflammation. Incorporation of highly sulfated glycosaminoglycans (GAGs) into collagen scaffolds has enabled the control of monocyte differentiation and macrophage phenotype *in vitro* (Kajahn et al., 2012). A reduction of pro-inflammatory cytokine secretion and an increase in anti-inflammatory cytokines were observed when primary macrophages were cultured with a collagen scaffold containing highly sulfated GAGs, in comparison with a collagen scaffold with either no GAGs incorporated or a less sulfated GAG incorporated. However, the application of this system in pre-clinical models is crucial to determine its potential usefulness in controlling macrophage phenotype in disease states.

A silk-fibroin/HA scaffold was used to treat post-MI inflammation. In addition to providing a structural support to the damaged myocardium, it was found that treatment with the composite silk-fibroin/HA reduced macrophage infiltration (CD68 positive cells) in comparison with a non-treated infarct (Chi et al., 2012). In addition, there was an increase in macrophages when bone marrow mesenchymal stem cells (BMSCs) were incorporated within the scaffold. The authors attribute this to the fact that the cells were obtained from male rats and transplanted in female rats. However, incorporation of the BMSCs improved myocardial function (wall thickness and fractional shortening), primarily through an increase in angiogenic factors and a resultant increase in vascular area. This emphasizes that reducing inflammation is an important factor, but that a multi-faceted approach is typically necessary to ensure adequate healing. Conversely, it was found in a separate study that a HA-based scaffold did not reduce inflammation unless it was coupled with MSCs (Muscari et al., 2013). This could be related to possible differences in the molecular weight of HA used. An increase in the vascular density was observed with MSC delivery, as with the previous study.

Delivery of ibuprofen from an electrospun, acid responsive poly (L-lactide) (PLLA) scaffold improved regeneration in a muscle wound model. Incorporation of sodium bicarbonate induced acid responsiveness of the PLLA scaffold, as it was observed that ibuprofen release was increased when the pH was changed from 7.4 to 5, whereas this property was not observed without the incorporation of the sodium bicarbonate. It was found that the ibuprofen reduced the inflammatory response as measured by immunohistochemical staining and assessment of gene expression of IL-6 and TNF-α, which resulted in improved muscle regeneration (Yuan et al., 2014). In a similar study, tetrandrine, an anti-inflammatory agent, was incorporated into a PLLA scaffold. It was found that this system could reduce the production of pro-inflammatory cytokines both *in vitro* and *in vivo* (Wang et al., 2014a). A subcutaneous study revealed that the tetrandrine-loaded scaffold had a reduced inflammatory response compared with the unloaded scaffold at 1, 4, and 12 weeks.

Delivery of *p*IL-10 was proposed as a mechanism by which to reduce the inflammatory response to porous poly (lactide-co-glycolide) (PLG) scaffolds. It was found that a macrophage cell line, RAW 264.7 cells, markedly reduced TNF-α and increased IL-10 expression when cultured in the presence of the PLG scaffolds, the IL-10 gene, and lipopolysaccharide (LPS) (Gower et al., 2014). This indicates a more anti-inflammatory nature of the macrophages following treatment with the IL-10 gene-loaded scaffold. Increased expression of IL-10 *in vivo* resulted in a reduction in leukocyte infiltration in the PLG scaffolds after 7 days. A decrease was also observed in the expression of interferon gamma (IFN-γ), but no difference was observed in the expression of IL-1β or TNF-α when compared with the control scaffolds. A follow-on study revealed that the mechanism behind this anti-inflammatory nature was a change in the phenotype of macrophages following treatment with the *p*IL-10 gene. It was found that transduction of RAW 264.7 macrophages with *p*IL-10 resulted in a switch toward a more regulatory macrophage phenotype, as well as preventing a shift toward a more inflammatory phenotype when in a pro-inflammatory environment (IFN-γ and LPS) (Boehler et al., 2014). A reduction in NF-kB activation was also observed following *p*IL-10 delivery, which was consistent with the reduction observed in TNF-α expression.

A sphingosine 1-phosphate receptor-3 agonist (FTY720) was used to modify the inflammatory response to a PLGA film. It was found that following implantation of a PLGA film, FTY720 delivery increased the arteriole and length density (Awojoodu et al., 2013). This was found to be as a result of an increase in the infiltration of anti-inflammatory monocytes to the site of implantation. *In vitro* studies revealed that FTY720 treatment changed the profile of inflammatory and regenerative cytokine secretion in both pro- and anti-inflammatory macrophages as well as human umbilical vein endothelial cells (HUVECs).

The examples discussed above (summarized in Table 1) provide an overview of the range of therapeutics that have been incorporated into solid biomaterial scaffolds. Therapeutics can be added to the scaffold immediately prior to implantation, or conjugated via linker systems. The incorporated therapeutics can help to overcome inflammation associated with the FBR, as well as any pre-existing pathological inflammation. The choice of therapeutic is dependent on the biomaterial itself, as well as the site into which it will be implanted. Thus, it is imperative to consider each pathology individually, rather than a ‘one size fits all’ approach. Furthermore, depending on the context of the injury, as well as the selected material, solid scaffold may be capable of providing mechanical support, i.e., in the case of bone scaffolds, or functions as a barrier in the case of dermal wounds.

**Table 1:** **Examples of anti-inflammatory therapies delivered from solid scaffolds**.

Biomaterial system	Therapeutic	Dose	*In vitro* characterization	*In vivo* model	*In vivo* outcome	Reference
PLGA	SDF-1α	50 μg SDF-1α	Stem cell migration assay	Mouse subcutaneous implantation	Increased vessel density	Thevenot et al. (2010)
					Enhanced MSC engraftment	
					Reduced inflammatory response (IL-1α, IL-6, TNF-α)	

Collagen	IL-10 pDNA	2.5 μg IL-10 pDNA	n/a	Rat subcutaneous model	Reduced cell infiltration	van Putten et al. (2009)
					Reduced EDI+ cells	
					Reduced collagenase activity	

Collagen	IL-10 pDNA polyplexes and MSCs	2 μg IL-10 pDNA	IL-10 secretion	Rat intramuscular implantation model	Increased stem cell survival	Holladay et al. (2011)
			Metabolic activity		Increased ratio of regulatory to inflammatory macrophages	

Collagen	IL-10 pDNA polyplexes and MSCs	2 μg IL-10 pDNA	n/a	Rat myocardial infarction model	Improved cardiac function and stem cell survival	Holladay et al. (2012)
					Increased ratio of regulatory to inflammatory macrophages	
					Reduction in apoptosis	

Silk-fibroin/hyaluronic acid	n/a	n/a	n/a	Rat myocardial infarction model	Reduced CD68+ cells	Chi et al. (2012)
					Improved wall thickness and fractional shortening	
					Reduced apoptosis	
					Increased vascular density, and VEGF, bFGF, and HGF expression	

Hyaluronic acid	MSCs	0.2–1 × 10^6^	MSC proliferations	Porcine myocardial infarction model	Reduced CD3+ cells	Muscari et al. (2013)
			VEGF expression		Reduced inflammatory score	

Poly (L-lactide)	Ibuprofen	Not specified	Ibuprofen release prolife in neutral and acidic pH	Rat muscle wound model	Improved muscle regeneration	Yuan et al. (2014)
			Fiber diameter
					Reduced IL-6 and TNF-α and IL-6 (protein and gene expression)	
					Increased VEGF and TGF-β (protein and gene expression)	

Poly (L-lactic acid)	Tetrandrine	5–20 mg/g	Tetrandrine release	Rat wound healing model	Reduction in inflammation in 20 mg/g tetrandrine group observed on H&E stained sections	Wang et al. (2014a,b)
			Cell viability	
			RAW 264.7 production of NO, TNF-α, and IL-6 on scaffold	
			Reduction in gene expression of iNOS, TNF-α, IL-6, and Cox-II	

Poly (lactide-co-Glycolide)	IL-10 viral vector	2 × 10^7^ viral particles	Reduced RAW 264.7 production of TNF-α and increased IL-10 following LPS treatment	Implantation into intraperitoneal mouse fat pad	Reduced leukocyte infiltration	Gower et al. (2014)
					Increased IL-10 expression and reduced IFN-γ expression	

Poly (lactic-co-Glycolic acid) film	FTY720	1:200 drug-to-polymer weight	Change in cytokine secretion of RAW 264.7 and HUVEC	Dorsal skinfold and muscle ischemia models	Increase in presence of anti-inflammatory macrophages	Awojoodu et al. (2013)
					Increased arteriole and length density	

### Hydrogels

One of the main reasons why anti-inflammatory therapy has become important in relation to biomaterials is to protect implanted cells and prevent rejection by the host. This can be elucidated with the example of islet cell delivery. Ideally, implanted islet cells will not be rejected by the body, and can produce insulin efficiently to correct diabetes. However, this is not the case in actuality, as the host often rejects the implanted cells and their function is compromised. Attempts to immuno-isolate cells using biomaterials have not proven successful, and thus anti-inflammatory signals are necessary to prevent rejection by the host immune system. Encapsulation in a biomaterial alone has not proved efficacious, and thus the incorporation of anti-inflammatory agent is the next logical step. Su et al. encapsulated islet cells in a PEG-based hydrogel, and conjugated an inhibitory peptide to the IL-1 receptor (IL-R1) (Su et al., 2010). This increased the survival of encapsulated cells *in vitro* following exposure to IL-1β, IFN-γ, and TNF-α, while islet cells were also able to continue glucose-stimulated release of insulin when incubated with β-cell specific T-lymphocytes.

Hyaluronic acid hydrogels have been extensively studied as materials to deliver anti-inflammatory therapies due to their biodegradable nature, as well as their own potential anti-inflammatory nature, which is dependent on the molecular weight chosen (Nakamura et al., 2004). Conjugation of dexamethasone to the HA was performed to increase drug retention by reducing diffusion from the hydrogel (Ito et al., 2007). *In vitro* studies revealed that the released dexamethasone reduced the expression of TNF-α and IL-6 from LPS-treated macrophages in a dose-dependent manner. Furthermore, *in vivo* studies showed that dexamethasone conjugated hydrogels had a reduced inflammatory response after 2 days when implanted subcutaneously. In a similar study, an anti-TNF-α antibody was conjugated to a HA hydrogel and applied to a burn wound (Friedrich et al., 2014). This treatment appeared to reduce the thickness of non-viable tissue, as well as IL-1β concentration and macrophage infiltration compared with the control.

Chitosan is a polysaccharide derived from crustaceans. It has been used for many biomedical applications (Khor and Lim, 2003; Jayakumar et al., 2010, 2007; Prabaharan, 2008). Specifically, it is commercially available as a bandage due to its clotting ability and its anti-bacterial properties (Chirkov, 2002). The addition of adipose-derived mesenchymal stem cells (ADSCs) to a chitosan hydrogel was seen to improve their survival in the infarcted myocardium (Liu et al., 2012). *In vitro* studies demonstrated the ROS scavenging properties of chitosan and its degradation products. MI was induced by permanent ligation of the coronary artery, followed by injection of the chitosan/ADSCs system. Treatment with the chitosan/ADSCs system resulted in a reduction in ROS, as observed by a reduction in dihydroethidium (DHE) staining. An improvement was observed at 4 weeks in ejection fraction and fractional shortening, while there was a reduction in apoptosis seen at 1 week. Additionally, there was a reduction in infarct size as well as an increase in wall thickness and vessel density in the infarct site. This study demonstrates the anti-ROS properties of chitosan, as it is hypothesized that chitosan enhances stem cell retention and survival in the myocardium (which was confirmed by *in vivo* bioluminescence imaging), partly due to its ability to scavenge ROS. This system was also utilized to treat ischemic injury in the kidney (Gao et al., 2012). A similar effect was observed, with a reduction in ROS expression, increased cell retention, and increased number of blood vessels.

A large-scale study was performed to identify a suitable anti-inflammatory drug for local immunosuppression of islet cells. From a large range of anti-inflammatory drugs, curcumin was identified as being capable of reducing inflammation and ensuing fibrosis following implantation of PLGA particles (Dang et al., 2013). Subsequent encapsulation of islet cells in alginate microcapsules, along with the identified anti-inflammatory curcumin, improved survival of the islet cells *in vivo*, with reduced fibrosis of the capsules and improved glycemic control in a chemically induced mouse type 1 diabetes model.

The anti-inflammatory drug resveratrol reduced inflammation in a cartilage defect model when delivered via a collagen hydrogel (Wang et al., 2014b). Resveratrol was grafted to poly (acrylic acid) and incorporated within a type I collagen hydrogel. Following delivery within a rabbit osteochondral defect model, treatment with resveratrol reduced inflammatory gene expression (IL-1β, MMP-13 and COX-2), with a resultant increase in bone and cartilage related genes (SOX-9, aggrecan, and collagen I and III). Gross and histological examination revealed the formation of cartilage-like neotissue, which compared favorably with no treatment and a collagen hydrogel without resveratrol.

A gelatin hydrogel was loaded with an anti-inflammatory peptide (triptolide) and BMP-2 to increase bone regeneration (Ratanavaraporn et al., 2012). It was found that incorporation of the anti-inflammatory peptide reduced the infiltration of inflammatory cells, except at the highest dose (10 mg), which indicates the importance of dose-response studies. Expression of IL-6, TNF-α, and NF-kB mRNA was also reduced. Reduced infiltration of inflammatory cells resulted in an increase in bone formation and bone mineral density, when accompanied by BMP-2 delivery. This study highlights the possibility of combining anti-inflammatory therapies with complementary therapeutics for the treatment of complex pathologies. In this case, addition of an osteoinductive protein, BMP-2, was essential for the formation of bone, while reduced inflammation serves to enhance this function.

A peptide nanofiber gel was loaded with dexamethasone to modify the inflammatory response (Webber et al., 2012).*In vitro* studies revealed that delivery of the dexamethasone from the peptide nanofiber gel reduced NF-kB activation in LPS treated THP-1 monocytes. An *in vivo* subcutaneous study revealed that in comparison with an unloaded peptide nanofiber gel, dexamethasone loaded gels reduced the presence of ROS and inflammatory cells after 3 and 21 days.

A PEG-maleimide hydrogel was developed with on-demand protease sensitive release of IL-1RA, the naturally occurring antagonist to the IL-1R. This system was used as a coating to reduce inflammation associated with implantation of neural electrodes (Gutowski et al., 2015). Immunofluorescent staining of inflammatory cell infiltration showed no difference between samples, while RT-PCR analysis showed minimal differences, with increases in IL-6, MMP-13, and ciliary neurotrophic factor (CNTF) the only changes detected. However, the gene expression analysis does allow for the specific-targeting of genes to reduce the inflammatory response in future studies.

Hydrogels have been utilized for the delivery of a range of anti-inflammatory therapeutics (see Table 2). Hydrogels have proven particularly attractive as a matrix to assist cell transplantation. They provide an ideal substrate for cell encapsulation and protection from the host response. By engineering a suitable matrix to encourage cell adhesion and proliferation, along with the incorporation of an anti-inflammatory agent to be released in the microenvironment, the survival of implanted cells can be promoted.

**Table 2:** **Examples of anti-inflammatory therapies delivered from hydrogels**.

Biomaterialsystem	Therapeutic	Dose	*In vitro* characterization	*In vivo* model	*In vivo* outcome	Reference
PEG hydrogel (10,000 Mw)	IL-1R inhibitory peptide (IL-1RIP)	1% IL-1RIP	Cell viability and insulin secretion from MIN6 cells	n/a	n/a	Su et al. (2010)

Hyaluronic acid	Dexamethasone	2.2–4.4 × 10^-5^ M	Cell viability	Subcutaneous implantation model	Reduced infiltration of macrophages and neutrophils observed on H&E stained sections	Ito et al. (2007)
			Hydrogel swelling ratio	
			Dexamethasone release profile	
			Reduced IL-6 and TNF-α production by RAW 264.7	

Hyaluronic acid	Anti TNF-α antibody	400 μg/ml	Binding affinity	Rat burn models	Reduced inflammatory cell infiltration observed on H&E stained sections	Friedrich et al. (2014)
					Reduced non-viable tissue	
					Reduced IL-1β protein expression	

Chitosan hydrogel	ADSCs	4 × 10^6^ ADSCs	Cell adhesion and expression of adhesion genes in response to ROS	Rat myocardial infarction model	Increased ICAM-1, VCAM-1, and SDF-1 expression and ADSC retention	Liu et al. (2012)
					Reduction in ROS, apoptosis, and infarct size	
					Increased wall thickness and blood vessel density, improved cardiac function	

Chitosan hydrogel	ADSCs	2 × 10^6^ ADSCs	n/a	Rat acute renal ischemia-reperfusion model	Reduction in apoptosis and ROS expression	Gao et al. (2012)
					Increased stem cell retention and renal cell proliferation	
					Reduction in serum levels of creatinine and blood urea nitrogen	

Alginate microcapsules containing islet cells	Curcumin	1 mg/ml	n/a	STZ-induced diabetic mouse model	Improved blood glucose level	Dang et al. (2013)
					Reduced gene expression of CD68, CD19, CD74, CD8, TNF-α, TGF-β, and αSMA	

Collagen hydrogel	Resveratrol	0.5%	Compressive strength free radical scavenging	Rabbit osteochondral defect	Reduction in IL-1β, MMP-13, and Cox-II mRNA	Wang et al. (2014a,b)
			Collagen degradation		Increased SOX-9, aggrecan, collagen I and III mRNA	
			Cell viability		Improved neotissue formation and integration	
					Increased collagen II deposition	

Gelatin	Triptolide [and BMP-2]	2.5, 5, or 10 mg	Release profile	Rat critical-sized bone defect	Reduced lymphocytes, netrophils, and mast cells	Ratanavaraporn et al. (2012)
			Relationship between degradation and release profile	
			Reduction in IL-6 and IL-10 protein expression in J774.1 macrophage-like cells		Reduced mRNA expression of IL-6, IL-10, TNF-α, NF-kB, and MMP-14	
			Proliferation and ALP activity in MC3T3-E1 cells		Increased bone mineral density	

Peptide amphiphile	Dexamethasone	Not specified	Dexamethasone release	Mouse subcutaneous model	Reduced ROS formation	Webber et al. (2012)
			Reduced NF-kB activity in THP-1 cells following LPS activation		Reduced inflammatory cell infiltration observed on H&E stained sections	
			Cell viability following treatment with Dex-PA	

Poly (ethylene glycol)-maleimide (Coating on a neural electrode)	IL-1RA	150 pg	Coating thickness	Rat neural implantation model	Increased IL-6, MMP-2, and CNTF	Gutowski et al. (2015)
			Cell adhesion	
			Reduced expression of IL-1β and TNF-α in microglia/astrocytes treated with GMCSF	
			IL-1RA release profile			

### Micro and nanoparticles

Nanoparticles have been widely used to deliver anti-inflammatory therapies. Whitmire et al. fabricated a new block copolymer that assembles into sub-micron particles and contains a moiety for tethering proteins (Whitmire et al., 2012). To this moiety, IL-1RA was conjugated. These particles were injected into the intra-articular joint space, where it was shown that they significantly enhanced the retention time of IL-1RA. However, to further increase retention time, the authors hypothesized that increased particle size could prove beneficial. To achieve this, a new self-assembling polymer composed of a polyhydroxyethylmethacrylate (pHEMA) backbone with a functionalized hydrophobic side chain of pyridine was fabricated, which allowed for variation in particle sizes (Singh et al., 2014). Particles of size 500 and 900 nm were fabricated and a fibronectin targeting ligand attached. Retention of BSA-loaded particles in the intra-articular space in the rat stifle joint was assessed using fluorescence imaging. It was found that the 900 nm particles were retained in the joint to a greater extent that either the soluble protein or the 500 nm particles. This emphasizes the importance of the design of appropriate and efficient systems to deliver therapeutics.

Polymer particles fabricated from poly (cyclohexane-1,4-diylacetone dimethylene ketal) (PCADK) were loaded with a p38 inhibitor to modulate the post-infarction inflammatory response in the myocardium (Sy et al., 2008). In an intramuscular model, the particles themselves were found to be non-inflammatory; while in an MI model, the particles significantly reduced superoxide and TNF-α production. This resulted in a reduction in fibrotic area as well as improved cardiac function. A similar study utilized the same particles to deliver superoxide dismutase (SOD) to the infarcted heart, which reduced superoxide expression and apoptosis (Seshadri et al., 2010). A subsequent improvement in cardiac function was also observed.

Encapsulating dexamethasone within PLGA particles reduced the *in vivo* inflammatory response to PLGA particles (Dang et al., 2011). It was found that encapsulation of dexamethasone within PLGA particles reduced both coverage of the PLGA particles with immune cells as well as cathepsin activity. Further investigation of the infiltration of inflammatory cells by examining histological sections revealed a reduction in the dexamethasone-loaded group compared with the unloaded control. Methylprednisolone (MP) was loaded within PLGA particles in an attempt to modify inflammation following injury of the spinal cord. The PLGA particles were suspended within an agarose gel to keep them in the local microenvironment of the injury (Chvatal et al., 2008). It was found that treatment with MP-loaded PLGA particles reduced the number of activated maicroglia, as well as the expression of the pro-inflammatory calpain and iNOS. This resulted in a reduction in lesion volume, indicating the potential of anti-inflammatory therapies in the spinal cord.

Delivery of siRNA targeting mitogen-activated protein kinase kinase kinase kinase 4 (MAP4K4) via β1,3-d-glucan particles suppressed systemic inflammation (Aouadi et al., 2009). Delivery of the siRNA orally resulted in a reduction in MAP4K4 gene expression in the lungs, liver, and spleen. In addition, when compared to a scrambled siRNA, MAP4K4 reduced the presence of LPS-induced TNF-α in both the serum and peritoneal fluid. The administration of MAP4K4 siRNA reduced LPS-induced lethality by inhibiting the expression of TNF-α and IL-1β. In a similar study using the same glucan particles, siRNA targeting TNF-α and osteopontin (OPN) was delivered systemically and localized to the macrophages present in the adipose tissue (Aouadi et al., 2013). It was found that silencing either TNF-α or OPN in the adipose tissue improved the glucose tolerance in ob/ob mice. Similarly, galactosylated trimethyl chitosanecysteine (GTC) nanoparticles were conjugated with MAP4K4 siRNA and delivered orally to treat ulcerative colitis (Zhang et al., 2013). It was found that this treatment reduced MAP4K4 and TNF-α mRNA, as well as TNF-α protein expression and MPO activity in the colon. Galactosylated particles showed increased efficacy compared with non-galactosylated nanoparticles, indicating the usefulness of targeting activated macrophages.

Utilization of PLGA particles to deliver PEI-conjugated FcγRIII-targeting siRNA to reduce inflammation proved efficacious in a rat model of temporomandibular joint (TMJ) inflammation (Mountziaris et al., 2012). Meal pattern analysis revealed an improvement following FcγRIII siRNA treatment, while a reduction was observed in the levels of two key pro-inflammatory cytokines, IL-1β and IL-6. Similarly, PLGA particles were used to delivery anti-TNF-α siRNA to treat inflammation associated with rheumatoid arthritis (RA) (Présumey et al., 2012). *In vitro* studies revealed that delivery of anti-TNF-α siRNA via PLGA particles could reduce LPS-induced TNF-α production in mouse monocytic J774 cells. Translation to an *in vivo* model of RA resulted in a reduction in TNF-α production and inflammation in the joint. Loading of PLGA particles with COX-2 siRNA and dexamethasone has also been proposed as a means by which to modify RA associated inflammation (Park et al., 2012). PLGA particles were loaded with dexamethasone, and then these drug-loaded particles had PEI/siRNA complexes attached to them. Pre-treatment of C28/I2 cells with TNF-α and IL-1β *in vitro* resulted in increased expression of COX-2 and iNOS. However, treatment with PLGA particles combined with dexamethasone and COX-2 siRNA reduced this induced expression. Interestingly, it was observed that dexamethasone had a minimal effect on inflammatory gene expression unless it was delivered via PLGA particles. This emphasizes the importance of the mode of delivery of an anti-inflammatory therapy, and suggests biomaterial systems as reservoirs of such therapeutics.

An annexin-A1 mimetic-peptide (Ac2-26) showed increased efficacy, when loaded within nanoparticles. Nanoparticles were fabricated from a biodegradable PLGA-b-PEG polymer using a nanoprecipitation method, with a collagen IV-targeting conjugate (Kamaly et al., 2013). These targeting nanoparticles loaded with Ac2-26 showed an increased ability to reduce the number of macrophages in an *in vivo* murine peritonitis model compared with non-targeting particles, srambled peptide-loaded particles, and also non-loaded particles. In an *in vivo* model of ischemia, targeted particles again compared favorably with scrambled-peptide loaded targeting particles and also non-targeting peptide loaded particles, with a reduction observed in myeloperoxidase activity. A similar formulation, but without the collagen IV-targeting conjugate, was used to form nanoparticles and deliver an anti-inflammatory synthetic liver X receptor agonist (GW3965) (Gadde et al., 2014). In peritoneal macrophages *in vitro*, it was shown that treatment with GW3965 in nanoparticle form reduced expression of monocyte chemoattractant protein 1 (MCP-1) and TNF-α. In an *in vivo* model of peritonitis, a reduction was observed in macrophage numbers, and MCP-1 and TNF-α gene and protein expression at a similar level to dexamethasone treatment.

A lipid nanoparticle was used to deliver a therapeutic siRNA that reduced the accumulation of pro-inflammatory monocytes to inflamed tissue (Leuschner et al., 2011). An siRNA targeting the chemokine receptor CCR2 was administered systemically, and shown to reduce the infarct size in an MI model, reduce inflammatory cells in atherosclerotic lesion, improve the survival of pancreatic islet allografts, and reduce tumor volume. This emphasizes the power of siRNA as a therapeutic modality capable of controlling cell phenotype, in this case pro-inflammatory macrophages.

Urethane acrylate non-ionomer (UAN) nanoparticles were functionalized with a targeting moiety to increase localization to tumors (Park et al., 2013). By conjugating an ICAM-1 targeting ligand to nanoparticles, localization to tumors and inflamed tissue was increased. UAN nanoparticles loaded with paclitaxel reduced the tumor volume compared without a targeting ligand. This shows the potential to target inflammation with nanoparticles and a targeting moiety. This system could be adapted to load various therapeutics, and also conjugated with different ligands for targeting of specific sights.

PLGA and chitosan were used to form bilayered nanoparticles for the delivery of two anti-inflammatory drugs, spantide II (SP) and ketoprofen (KP) (Shah et al., 2012). These nanoparticles were combined with a skin-permeating nanogel, and used in two models of allergic contact dermatitis (ACD) and psoriatic plaque. Ear thickness was reduced in the ACD model, while trans-epidermal water loss was reduced in the psoriatic model, the two primary endpoints in both models, indicating the power of anti-inflammatory delivery via nanoparticles.

Particulate systems, whether in the micro or nano range, are versatile delivery systems capable of delivering drugs, proteins, and nucleic acids, or combinations thereof (see Table 3). However, issues with retention at the inflamed site persist, amplified by the fact that activated macrophages clear particles in a size and shape-dependent manner (Champion and Mitragotri, 2006). Thus, choice of particle size/shape is imperative, as well as other strategies to avoid uptake and clearance. These may include tethering of “self” peptides to particles (Rodriguez et al., 2013), or combination with solid or hydrogel scaffolds to increase bulk retention in the target tissue.

**Table 3:** **Micro and nanoparticles used to deliver anti-inflammatory therapies**.

Biomaterialsystem	Therapeutic	Dose	*In vitro* characterization	*In vivo* model	*In vivo* outcome	Reference
Tetraethylene glycol and cyclohexyl methacrylate nanoparticles	IL-1RA	5 μg IL-1RA	Nanoparticle size	Rat intra-articular model	Increased retention of IL-1RA compared with saline delivery	Whitmire et al. (2012)
			Target specificity	
			NF-κβ activity in NIH3T3 fibroblasts	

Poly (hydroxy-ethyl-methacrylate) [p(HEMA)]	(BSA as a model protein)	500 μg Vivo-Tag-S750-BSA	Particle size Cell viability	Rat intra-articular injection	Increased retention compared with soluble protein	Singh et al. (2014)

Poly (cyclohexane- 1,4-diylacetone dimethylene ketal) (PCADK)	P38 inhibitor	50 μg P38 inhibitor	Particle size Activation of RAW 264.7 macrophages	Rat myocardial infarction model	Reduced P38 activation, superoxide, and TNF-α production	Sy et al. (2008)
					Reduced fibrotic area and improved cardiac function	

Poly (cyclohexane- 1,4-diylacetone dimethylene ketal) (PCADK)	Superoxide dismutase (SOD)	80U SOD	Particle size	Rat myocardial infarction model	Reduced superoxide production and apoptosis	Seshadri et al. (2010)
			Superoxide scavenging		Improved cardiac function	

PLGA	Dexamethasone	1.3 and 26 wt%	Dexamthasone loading and release	Mouse subcutaneous injection	Reduced cathepsin activity up to 10 days	Dang et al. (2011)
					Reduced inflammatory cell infiltration up to 30 days	

PLGA	Methylprednisolone	156 μg	Methylprednisolone release	Rat spinal cord contusion model	Reduced ED1+ cells	Chvatal et al. (2008)
			NO production by LPS-treated microglia		Reduced Calplain and iNOS	
					Reduced lesion volume	

β1,3-d-glucan	MAP4K4 siRNA	20 μg/kg body weight	MAP4K4 and TNF-α knockdown	Mouse LPS-induced lethality	Reduced MAP4K4 mRNA in peritoneal macrophages, spleen, liver, and lung	Aouadi et al. (2009)
					Reduced TNF-α and IL-1β mRNA	
					Reduced serum and peritoneal TNF-α	

Galactosylated Trimethyl Chitosanecysteine	MAP4K4 siRNA	250 μg/kg body weight/day	Charge and cell uptake	Mouse ulcerative colitis	MAP4K4 and TNF-α mRNA knockdown	Zhang et al. (2013)
			MAP4K4 and TNF-α mRNA knockdown		Reduced colonic TNF-α protein and MPO activity	
			Reduced TNF-α protein expression		Reduced infiltration of mononucleur cells observed on H&E sections	

PLGA	FcγRIII-targeting siRNA	≈16–23 μg	siRNA release	Rat temporomandibular inflammation	Reduced IL-1β and IL-6 protein expression	Mountziaris et al. (2012)
			siRNA loading efficiency		Reduced FcγRIII expression	

PLGA	TNF-α siRNA	0.12 nM	Particle size siRNA release	Mouse collagen induced arthritis	Reduced synovial inflammatory score	Présumey et al. (2012)
			Reduced TNF-α mRNA and protein expression		Reduced TNF-α protein expression	

PLGA	Dexamethasone and COX-2 siRNA	n/a	Dexamethasone and siRNA loading	n/a	n/a	Park et al. (2012)
			Particle size, charge, cell viability, uptake, and transfection efficiency	
			PGE_2_ secretion	
			COX-2 and iNOS knockdown in C28-I2	
			Reduced mPGES-1, COX-2, and iNOS protein expression	
			Reduced caspase-3	

PLGA-b-PEG	Ac2-26 (annexin-A1 mimetic peptide)	100 ng	Particle size	Murine peritonitis	Reduced number of PMNs	Kamaly et al. (2013)
			Ac2-26 release	Muscle ischemia	Reduced MPO activity	
PLGA-b-PEG	GW3965 (liver X receptor agonist)	8 mg/kg	Particle size	Mouse zymosan-induced Peritonitis	Reduced PMN infiltration	Gadde et al. (2014)
			GW3965 release profile		Reduced TNF-α and MCP-1 gene and protein expression in peritoneal exudates	
			Reduced TNF-α and MCP-1 gene and protein expression in peritoneal macrophages	

C12-200 lipid, disteroylphosphatidyl choline, cholesterol, and PEG-DMG	CCR2 siRNA	1 mg/kg	n/a	Mouse ischemia/reperfusion	Reduced CCR2 expression	Leuschner et al. (2011)
				Mouse permanent ligation	Reduced myocardial area-at-risk	
				Mouse streptozotocin-induced diabetes	Reduced Ly-6Cigh^high^ macrophages, CD11b+ cells, and lesion volume in atheresclerotic plaque	
				Mouse islet transplantation	Increased survival of pancreatic islet allografts	
				Mouse tumor xenograft model	Reduced tumor volume, tumor associated macrophages, and CD11b+ cells	

Urethane acrylate non-ionomer (UAN)	Paclitaxel	200 μg UAN-Paclitaxel nanoparticles	Viability and dose response	Mouse tumor cytotoxicity assay	Reduced tumor volume	Park et al. (2013)

PLGA and chitosan	Spantide II and ketoprofen	Not specified	Particle loading efficiency	Mouse allergic contact dermatitis	Increased drug retention	Shah et al. (2012)
			Spantide II and ketoprofen release profile		Reduced ear	
					Reduced IL-17 and IL-23 expression	
					Reduced trans-epidermal water loss	

## Future Directions

Modulation of inflammation is a key component for the success of biomaterial and tissue engineering based strategies. This is the case both in terms of modulating the FBR to ensure the survival and functionality of implanted devices and also in delivering anti-inflammatory therapeutics to sites of pathological inflammation. Thus, strategies to improve the efficacy of anti-inflammatory therapies are vital.

**Figure 2:**
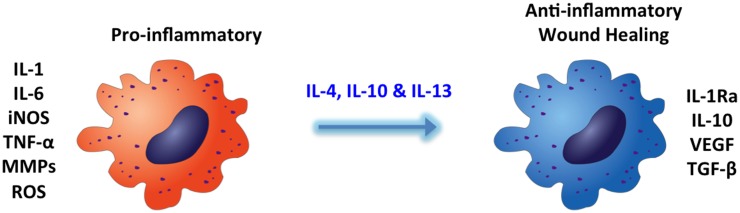
**Macrophage phenotype: inflammation may be controlled by modulation of the phenotype from a pro-inflammatory nature to anti-inflammatory and wound healing**. Cytokines including IL-4, IL-10, and IL-13 have been implicated in the transition of macrophages from a pro-inflammatory phenotype to a more regulatory, anti-inflammatory and wound healing phenotype *in vivo*.

The inflammatory response is often quantified in terms of the number of macrophages present, the phenotype of these macrophages, or both. Thus, the macrophage is and has been identified as a key component of inflammation (Wynn and Barron, 2010; Koh and DiPietro, 2011) and the FBR to biomaterials (Xia and Triffitt, 2006; Brown et al., 2012). Furthermore, where along the spectrum of macrophage activity, these macropahages are a key determinant in whether inflammation will become resolved in a satisfactory manner (Mosser and Edwards, 2009). Thus, control over macrophage can be key to modulating inflammation and resolving it. Cytokines such as IL-4, IL-10, and IL-13 (see Figure 2) have been identified as playing a role in shifting the balance from a pro-inflammatory macrophage to that of a macrophage more anti-inflammatory in nature, promoting tissue repair and remodeling (Mantovani et al., 2013). A number of biomaterial systems have been developed to deliver pDNA to macrophages (Helary et al., 2012; Mahor et al., 2012). Thus, specific delivery of molecules to direct macrophages toward an anti-inflammatory phenotype rather than a pro-inflammatory phenotype holds promise as a treatment for inflammation.

A number of natural biomaterials have intrinsic anti-inflammatory properties, including HA and chitosan. Thus, they are suitable as carriers for anti-inflammatory therapeutics. However, synthetic materials are also capable of acting in an anti-inflammatory way. Dead and dying cells release their contents, including nucleic acids, both RNA and DNA. These nucleic acids act as DAMPs and activate toll like receptors (TLRs), stimulating an inflammatory response. It was found that binding of nucleic acids produced by injury by cationic polymers reduces associated inflammation, both *in vitro* and *in vivo* (Lee et al., 2011). This ability to act as a molecular scavenger for nucleic acids could be adapted for other molecules, with a biomaterial acting as a “sponge” to scavenge and absorb pro-inflammatory cytokines in an inflamed environment. However, a more specific approach, possibly involving conjugation of pro-inflammatory targeting antibodies, would be necessary to avoid the uptake of all cytokines and growth factors. In a similar way, materials may be protected from an inflammatory response by the conjugation of immunomodulatory “self” proteins. Attachment of CD200 protein to a biomaterial surface reduced the production of TNF-α and IL-6 *in vitro* in response to LPS and IFN-γ stimulation, by activating pathways that inhibit inflammation (Kim et al., 2014). This anti-inflammatory nature was confirmed *in vivo* using bioluminescence imaging to detect ROS activity. In comparison with unmodified materials, the CD200 modified material displayed a drastically reduced tissue response. Strategies such as this may reduce inflammation in the context of implantable devices, but may also be used as a means by which to reduce inflammation locally in a pathological condition.

Nanoparticles can be used to deliver multiple therapeutics. Codelivery of IL-10 siRNA and a pDNA vaccine using PLGA-PEI nanoparticles were used to modify the Th1 to Th2 balance during immunotherapy (Singh et al., 2008). Similarly, lipid-like nanoparticles were used to simultaneously deliver both pDNA and siRNA (Dong et al., 2014). *In vivo* studies showed the potential to upregulate luciferase activity and reduce Tie-2 expression using a luciferase pDNA, and a Tie-2 targeting siRNA. Selection of different nucleic acids creates an opportunity to upregulate an anti-inflammatory mediator while knocking down a pro-inflammatory mediator concurrently. This may prove key as inflammation is a complex phenomenon that requires multiple points of control.

Responsive systems are a well-utilized facet of biomaterial design. In order to design biodegradable systems, crosslinkers with MMP-cleavable sequences are incorporated into typically non-degradable sequences. In this way, MMP sensitivity is built into the system. Therapeutics may also be conjugated to biomaterials using sensitive linker systems. That is, the therapeutic will be released in response to a pre-determined stimulus. For example, previously therapeutics have been conjugated to a fibrin scaffold such that they would only be released by MMP activity, resulting in improved efficacy (Zisch et al., 2003; Ehrbar et al., 2004). ROS sensitivity has been identified as a key mediator for the release of therapeutics (Yoshitomi and Nagasaki, 2014). Nanoparticles composed of β-cycoldextrin were engineered with ROS sensitivity, and tested *in vitro* and *in vivo*. ROS responsive-release of docetaxal from nanoparticles *in vivo* was assessed as a means by which to reduce tumor volume (Zhang et al., 2015).

As stated, inflammation is a key determinant in the FBR and how it progresses. In addition, in plays a key role in the progression of a number of pathological conditions in which it is dysregulated. However, in most cases, dysregulation of and excessive inflammation is not the only process involved. For instance, in the case of diabetic wound healing, inflammation is excessive, but there are also issues with insufficient angiogenesis as well as ECM turnover. Thus, approaches that aim to reduce inflammation and also modulate other processes are needed. Biomaterial systems can be designed to load and release multiple, complementary therapeutics (Browne and Pandit, 2014). A multi-modal system composed of fibrin microspheres in a fibrin hydrogel was used to modify inflammation and angiogenesis, through the delivery of two pDNAs encoding Rab18 and endothelial nitric oxide synthase (eNOS) (Kulkarni et al., 2014). This improved healing in a diabetic rabbit wound model. A similar system composed of collagen spheres-in-hydrogel was used to deliver IL-10 and eNOS pDNA in a staggered manner *in vitro* (Alexander et al., 2013), while *in vivo* assessment of dual delivery of anti-inflammatory and pro-angiogenic peptides showed a combinatorial effect (Zachman et al., 2012).

## Outlook

Control over inflammation is vital for the success of biomaterial and tissue engineered therapies. Modification of inflammation is key to reducing the impact of the FBR. In addition, biomaterials also offer the opportunity to control inflammation in pathological conditions, through increased localization and retention of therapies in the inflamed microenvironment. The form of biomaterial used (solid scaffold, hydrogel, or micro/nanoparticle) is dependent on the target site, with considerations such as ease of access and retention paramount. In addition, the choice of anti-inflammatory therapeutics is typically dependent on the cause of inflammation.

Future therapeutics will focus on control of macrophage phenotype, the use of anti-inflammatory materials, complementary combinations of anti-inflammatory therapeutics, and biomaterial systems that release anti-inflammatory therapeutics in response to inflammatory stimuli, such as ROS and MMPs.

## Conflict of Interest Statement

The authors declare that the research was conducted in the absence of any commercial or financial relationships that could be construed as a potential conflict of interest.
